# Neuronal Migration and Ventral Subtype Identity in the Telencephalon Depend on SOX1

**DOI:** 10.1371/journal.pbio.0030186

**Published:** 2005-05-17

**Authors:** Antigoni Ekonomou, Ilias Kazanis, Stavros Malas, Heather Wood, Pavlos Alifragis, Myrto Denaxa, Domna Karagogeos, Andrew Constanti, Robin Lovell-Badge, Vasso Episkopou

**Affiliations:** **1**Mammalian Neurogenesis Group, MRC Clinical Sciences CentreImperial College School of Medicine, Hammersmith Hospital Campus, LondonUnited Kingdom; **2**Medical School and Institute of Molecular Biology and Biotechnology, University of CreteHeraklionGreece; **3**Department of Pharmacology, The School of PharmacyLondonUnited Kingdom; **4**Division of Developmental Genetics, National Institute of Medical ResearchLondonUnited Kingdom; Columbia UniversityUnited States of America

## Abstract

Little is known about the molecular mechanisms and intrinsic factors that are responsible for the emergence of neuronal subtype identity. Several transcription factors that are expressed mainly in precursors of the ventral telencephalon have been shown to control neuronal specification, but it has been unclear whether subtype identity is also specified in these precursors, or if this happens in postmitotic neurons, and whether it involves the same or different factors. SOX1, an HMG box transcription factor, is expressed widely in neural precursors along with the two other SOXB1 subfamily members, SOX2 and SOX3, and all three have been implicated in neurogenesis. SOX1 is also uniquely expressed at a high level in the majority of telencephalic neurons that constitute the ventral striatum (VS). These neurons are missing in *Sox1*-null mutant mice. In the present study, we have addressed the requirement for SOX1 at a cellular level, revealing both the nature and timing of the defect. By generating a novel *Sox1*-null allele expressing β-galactosidase, we found that the VS precursors and their early neuronal differentiation are unaffected in the absence of SOX1, but the prospective neurons fail to migrate to their appropriate position. Furthermore, the migration of non-*Sox1*-expressing VS neurons (such as those expressing *Pax6*) was also affected in the absence of SOX1, suggesting that *Sox1*-expressing neurons play a role in structuring the area of the VS. To test whether SOX1 is required in postmitotic cells for the emergence of VS neuronal identity, we generated mice in which *Sox1* expression was directed to all ventral telencephalic precursors, but to only a very few VS neurons. These mice again lacked most of the VS, indicating that SOX1 expression in precursors is not sufficient for VS development. Conversely, the few neurons in which *Sox1* expression was maintained were able to migrate to the VS. In conclusion, *Sox1* expression in precursors is not sufficient for VS neuronal identity and migration, but this is accomplished in postmitotic cells, which require the continued presence of SOX1. Our data also suggest that other SOXB1 members showing expression in specific neuronal populations are likely to play continuous roles from the establishment of precursors to their final differentiation.

## Abstract 2

The mechanisms whereby neurons establish their final identity are largely unknown. This study shows that the transcription factor SOX1 plays an important part in this process.

## Introduction

The telencephalon is subdivided into dorsal (pallial) and ventral (subpallial) territories, which give rise to the cerebral cortex and the underlying basal ganglia, respectively. The embryonic subpallium consists of large protrusions—the ganglionic eminences. Several distinct types of neurons originate in the ganglionic eminences, and some migrate as far as the olfactory bulb, hippocampus, and neocortex [1–3], while others contribute more locally. The majority of neurons of the lateral ganglionic eminence (LGE) form the dorsal and ventral striatum (VS). The VS includes the caudate, putamen, nucleus accumbens, and olfactory tubercle (OT), which control various aspects of motor, cognitive, and emotional functions [4,5]. Little is known about the molecular mechanisms that control the emergence of various groups of neurons with distinct identities in this region.

Gene-expression studies and loss-of-function mutations in homeodomain transcription factors such as PAX6 [6,7] and GSH2/1 [8–13] confirm fate-mapping findings [14–16] that the majority of the VS neurons are specified within the progenitor domain of the LGE. The proneural basic helix-loop-helix (bHLH) factor MASH1 also marks the precursors of early-born neurons in the LGE progenitor domain, and its loss in the mouse leads to a deficit of both precursors and neurons of the telencephalon, including loss of VS neurons [17,18]. Therefore, GSH2 and MASH1 control VS precursor patterning and specification, but as they are not expressed in postmitotic cells it remained unknown to what extent they are involved in the emergence of neuronal subtypes in the ventral telencephalon, and whether different transcription factors with neuron-specific expression are required.

The SOX proteins constitute a family of transcription factors [19,20] that regulate transcription through their ability to bind to specific DNA sequences via their HMG box domains [21–24]. There are 20 *Sox* genes in mammals, and at least half are expressed in the developing nervous system [20,24]; however, their role in neural development is poorly understood. SOX1, SOX2, and SOX3 constitute the SOXB1 subfamily and share more than 95% identity within their HMG boxes and significant homology outside [25,26]. All three proteins are expressed in the neuroepithelium throughout central nervous system (CNS) development [25,27], and as they tend to be down-regulated upon neural differentiation they have been used as markers for neural stem cells and precursors [28,29]. Several studies suggest that SOXB1 factors function in stem cells and precursors to maintain broad developmental potential [30] and neural stem cell identity [30–32] by counteracting neurogenesis. Contradictory evidence, however, suggests that SOX1 promotes neurogenesis and cell cycle exit [33]. However, mice that are null for *Sox1* [34] or *Sox3* [35], or mice with one *Sox2* allele deleted and the other hypomorphic [36], exhibit phenotypes associated with the loss of or functional deficit of only specific neuronal populations. As these SOXB1 factors are expressed in both precursors and neurons that are affected in these mutant mice, it was not known whether their function is required in precursors, postmitotic cells, or both.

We have previously shown that SOX1 is essential for the terminal differentiation of lens fibers and the activation of γ-crystallins [37], and for the development of VS neurons, the lack of which is associated with epilepsy [34]. Here, we show that absence of SOX1 has no effect on the generation, proliferation, and patterning of neuronal precursors. This is probably due to functional compensation by SOX2 and SOX3, which are co-expressed with SOX1 in precursors. Moreover, mice lacking only the neuron-specific expression of *Sox1* in the ventral telencephalon still fail to develop VS neurons, revealing its requirement within these neurons. Consistent with this, maintenance of *Sox1* expression in neurons of the ventral telencephalon is sufficient to direct them to the VS, confirming the adequacy of SOX1 function in postmitotic cells for their migration and identity. Therefore, VS-specific neuronal migration and subtype identity most likely is initiated in precursors but is completed in postmitotic cells by transcription factors such as SOX1.

## Results

### SOX1 Is Essential for the Histogenesis of the VS

**Figure 1:**
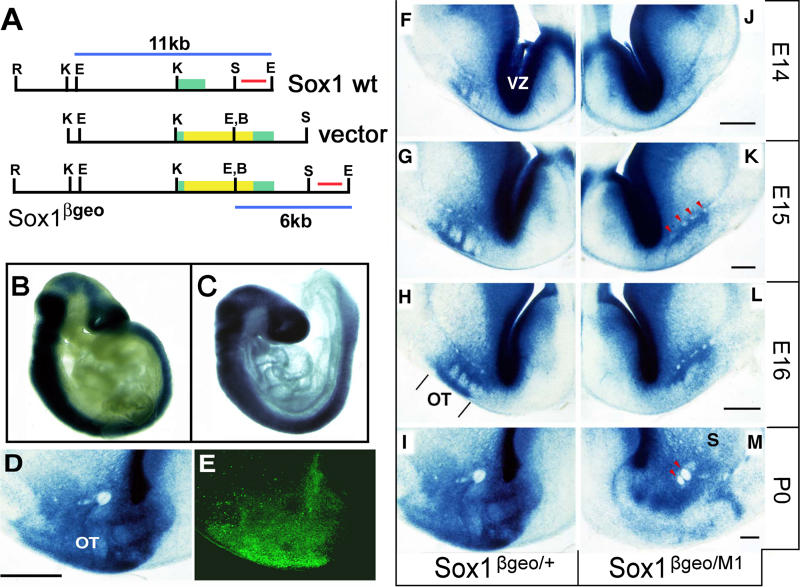
The Mouse *Sox1* ^βgeo^ Allele Reveals the Requirement of SOX1 in the Development of VS Neurons (A) Strategy for targeting of the *Sox1* locus by insertion of β*geo.* Restriction enzymes: RV, EcoRV; K, KpnI; E, EcoRI; S, SpeI; B, BamHI. Yellow boxes indicate β*geo*, green, SOX1 exon, and blue lines indicate fragments appearing in Southern blots of EcoR1-digested genomic DNA, hybridized with the external probe, which is shown with red lines. (B–E) X-gal and SOX1 antibody staining of *Sox1* ^βgeo/+^. Comparison of *Sox1* ^β*geo*^ expression visualized by X-gal staining (B and D) and the endogenous wild-type *Sox1* gene visualized by whole-mount in situ (C) and SOX1 antibody staining (E). (B and C) show E9-stage embryos and (D and E) show coronal sections of newborn ventral telencephalon. (F–M) 100-μm coronal sections (Vibratome) were stained with X-gal to identify cells with *Sox1* promoter activity*.* (F–I) show *Sox1* ^βgeo/+^ forebrain sections from E13 to birth (P0) showing normal migration of *Sox1*-expressing cells from the VZ to the site of the OT, including striatal bridges. (J–M) show sections of *Sox1* ^βgeo/M1^ forebrain, showing absence of X-gal staining in the OT and the striatal bridges. Red arrowheads show the anterior commissure. Scale bar = 500 μm for (B) and (C) and 300 μm for (D–M).

To generate a detailed map of *Sox1* expression in the developing and adult brain of mice, and to perform comparative studies between homozygotes and heterozygotes, we generated a novel targeted allele referred to as *Sox1* ^βgeo^. This contains an insertion of β-galactosidase-neo (β*geo*) fusion protein in-frame with the SOX1 open reading frame (Figure 1A). Mice homozygous for *Sox1* ^βgeo^ are null for SOX1 and exhibit the same phenotype as the previously described mice, which carry a deletion of the SOX1 coding region (*Sox1* ^M1^) [34,37], namely, lens defects and epileptic seizures. Staining for β-galactosidase activity (X-gal staining) in *Sox1* ^βgeo/+^ heterozygous embryos matches that for the wild-type allele as revealed by whole-mount in situ hybridization and SOX1 antibody staining (Figure 1B–1E).

To elucidate the role of SOX1 in the formation of the VS, we compared the expression pattern of *Sox1* in heterozygous (Figure 1F–1I) and homozygous (Figure 1J–1M) brains from embryonic day 14 (E14) to postnatal day 0 (P0) using X-gal staining. Throughout much of the CNS, X-gal staining in the *Sox1* ^βgeo/βgeo^ homozygotes is double the intensity observed in heterozygotes (data not shown). To perform comparative histological studies, we equalized the levels of X-gal staining in homozygous mice with those of heterozygous animals by generating homozygous mice that harbor two different *Sox1*-null alleles: β*geo* (*Sox1* ^βgeo^) and the previously described M1-targeted allele (*Sox1* ^M1^) [37], which does not express β-galactosidase.

**Figure 2:**
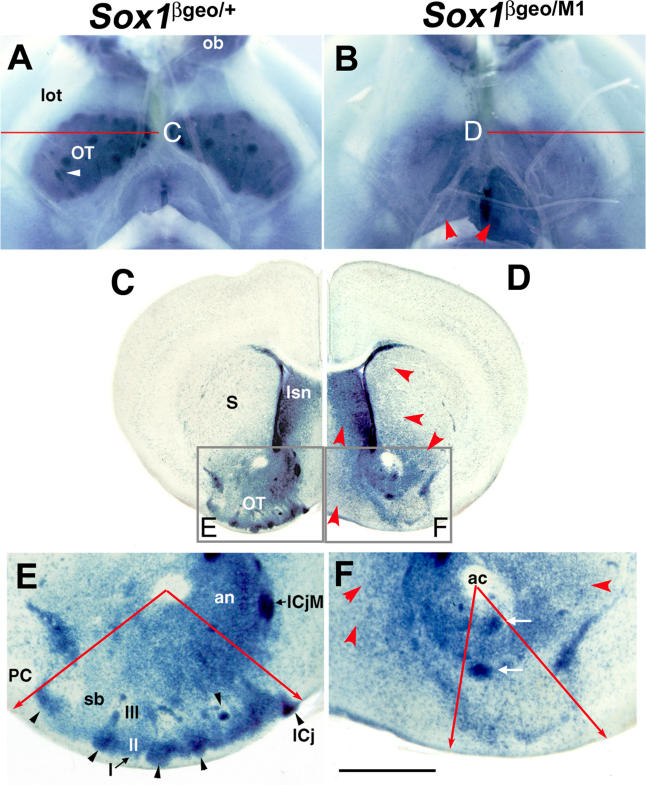
Ectopic Distribution of *Sox1*-Null Neurons X-gal staining of mouse forebrains at P16. (A and B) show intact forebrain viewed from the ventral surface, and (C–F) show 150-μm coronal Vibratome sections for *Sox1* ^βgeo/+^ mice (A, C, and E) and *Sox1* ^βgeo/M1^ mice (B, D, and E). Red arrows indicate the width of the OT. Red arrowheads indicate increased X-gal staining at more medial and posterior areas of the brain in (B), and in the striatum and septum in (D) and (F). White arrowheads indicate islands other than the medial islands of Calleja. an, accumbens nucleus; I, II, III, cell layers of the OT; ICjM, medial islands of Calleja; lot, lateral olfactory tract; lsn, lateral septal nucleus; ob, olfactory bulb; PC, olfactory (piriform) cortex; S, striatum; sb, striatal bridge Scale bar = 500 μm.

Our analysis shows (via X-gal staining) that in the developing forebrain, *Sox1* is expressed throughout the ventricular zone (VZ) and subventricular zone (SVZ) and in neurons around the anterior commissure region, where the prospective nucleus accumbens forms (red arrowheads in Figure 1K and 1M), and in the striatal bridges that link this intermediate cluster of cells with the prospective OT region toward the pial surface. X-gal-positive neurons start populating the OT area as early as E14 and continue to accumulate at least until birth (Figure 1F–1I). In the *Sox1* ^βgeo/M1^-null mutants, the X-gal staining pattern of the VZ/SVZ is indistinguishable from that of the *Sox1* ^βgeo/+^ heterozygotes, and there is no obvious deficit of X-gal-positive cells around the anterior commissure. On the other hand, both the striatal bridges and the OT layers are absent in the *Sox1*-null brain at all developmental stages (compare Figure 1F–1I to 1J–1M). It is unlikely that neurons die en masse in this region, because an apoptosis assay did not reveal any evidence of increased cell death in the mutant (data not shown). In addition, X-gal staining is increased throughout the ventral telencephalon in the mutant postnatal brain (red arrowheads in Figure 2), suggesting that the *Sox1*-null cells are not correctly specified and contribute to other brain regions. Interestingly, although neurons that form the core of the nucleus accumbens express *Sox1* highly, they form normally (Figure 2F and red arrowheads in Figure 1) and do not depend on SOX1 for their development. Therefore, SOX1 is required for histogenesis of the OT throughout its development.

### Normal Precursor Proliferation and Neurogenesis but Loss of OT Neuronal Differentiation in the Absence of SOX1

**Figure 3:**
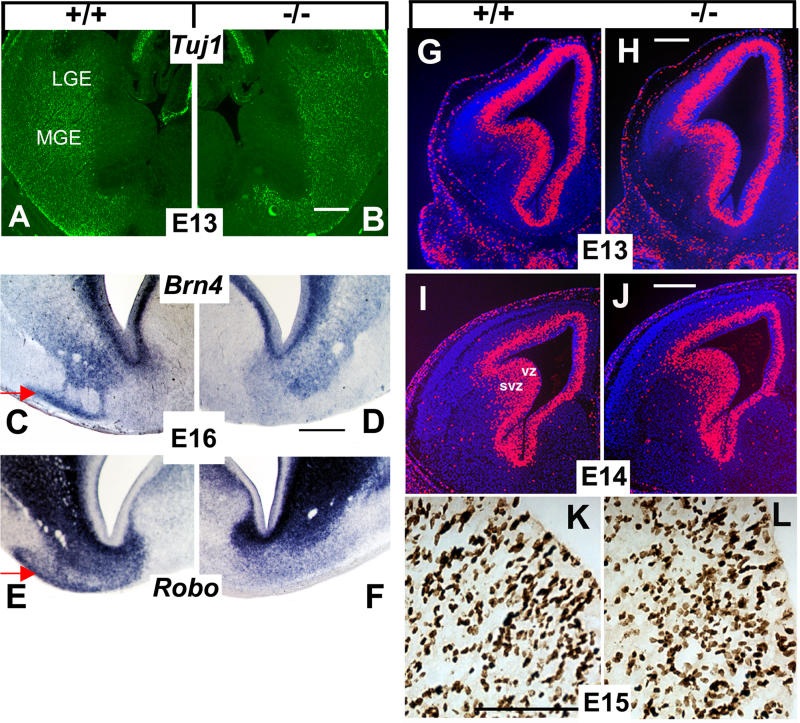
Normal Precursor Proliferation and Neurogenesis but Loss of OT Neuronal Differentiation in the Absence of SOX1 Coronal brain sections from the ventral telencephalon of wild-type (+/+) and *Sox1*-null (−/−) embryos. TuJ1 immunolabeling (A and B) at E13 shows no difference in early neuronal differentiation embryos; in situ hybridization at E16 for *Brn4* (C and D) and *Robo* (E and F) shows absence of differentiation in the mutant at the prospective OT area. Red arrow in wild-type brain sections indicates OT. Telencephalic sections of wild-type (G, I, and K) and *Sox1*-null mutant (H, J, and L) embryonic brains were harvested 1 h after BrdU injection at E13 (G and H), E14 (I and J), and E15 (K and L) to detect actively dividing cells of the VZ/SVZ. Positive cells were visualized with anti-BrdU immunofluorescence (G–J) or with DAB staining (K and L). (K and L) show dorsal LGE area at high magnification. No differences were detected in the proliferation precursors at all stages examined, and no ectopic proliferation was observed in the mutant brains*.* Measurements and statistical analysis of BrdU-positive cells were performed on the DAB-stained sections, showing no significant differences (see Table S1). Scale bar = 300 μm (A and B), 300 μm (C–F), 500 μm (G–J), 500 μm (K and L).

Studies with conflicting results suggest that SOX1 either, like SOX2 and SOX3, counteracts neurogenesis [32] or, unlike SOX2 and SOX3, promotes neurogenesis [33]. To examine whether the loss of SOX1 affects general neural differentiation in the area of the striatum, we used an anti-βIII-tubulin (TuJ1) antibody*,* which is a marker for immature neurons [38], at E13, a critical time of differentiation in the LGE. TuJ 1 immunocytochemistry did not reveal any obvious general differentiation problems in the *Sox1* mutants (Figure 3A and 3B), suggesting that loss of SOX1 alone is not sufficient to compromise general neuron differentiation and maturity. The differentiation and distribution of specific mature neurons was examined in our previous study at adult stages with the expression of striatal markers such as *preproenkephalin* and *Gad65/67* [34], and in our current study, at embryonic stages with additional markers such as *Brn4* [39] (Figure 3C and 3D) and *Robo* [40] (Figure 3E and 3F). This analysis revealed a differentiation defect restricted in the region of the nucleus accumbens/OT, and not the rest of the striatum.

As *Sox1* expression is associated with dividing cells throughout the neuroepithelium, we examined whether a proliferation defect could partially account for the cell deficit in the ventral telencephalon. We used 5-bromo-2′-deoxyuridine (BrdU) to label all proliferating precursors in wild-type and *Sox1*-null embryos at E13–E15, and harvested their brains 1 h later. Most of the dividing cells were found in the VZ/SVZ, and there was no increase or ectopic proliferation (Figure 3G–3L; Table S1). Therefore, SOX1 is unlikely to be required for proliferation or the exit of precursors from the cell cycle in the LGE. Collectively, the above data show that SOX1 is not essential for the proliferation of precursors and general neuronal differentiation. However, it is required specifically for the differentiation and/or migration of VS neurons.

### Early- and Late-Born OT Neurons Fail to Migrate in the Absence of SOX1

**Figure 4:**
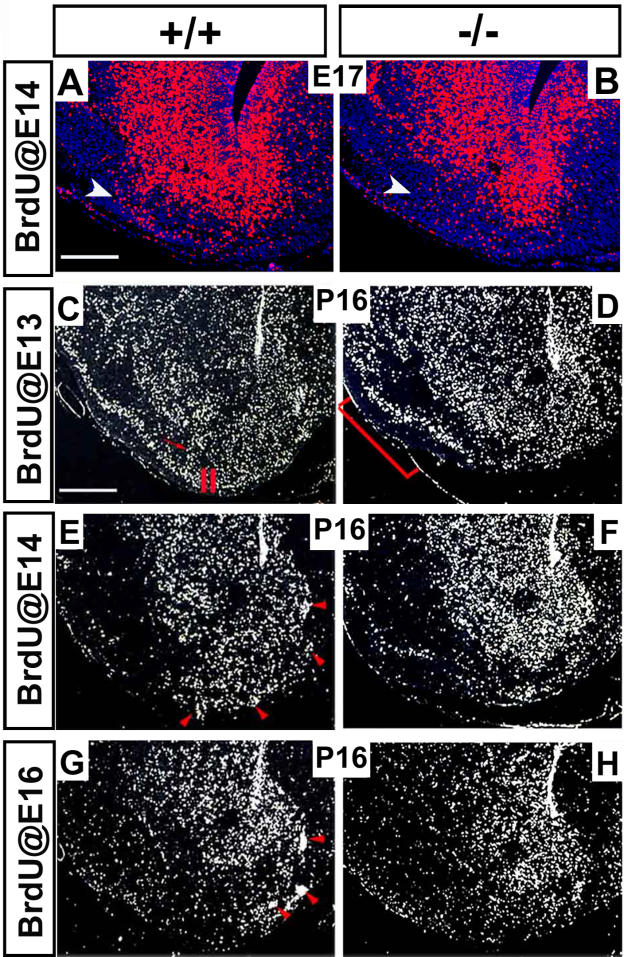
Failure of Neurons to Migrate to the VS in the Absence of SOX1 This figure shows BrdU labeling of proliferating cells in the developing forebrain. Immunohistochemistry was performed on 5-μm coronal sections, cut at the level of the OT. (A and B) Sections at E17, after BrdU injection at E14. White arrowheads in (A and B) indicate streams of migrating cells. (C–H) Sections at P16, after BrdU injection at E13 (C and D), E14 (E and F), or E16 (G and H). The DAB reaction product (C–H) was viewed under dark-field illumination. “II” is layer II of the OT, and the red bracket indicates the olfactory cortex. Note E13-born neurons contribute laterally to the olfactory (piriform) cortex, and medially to the layer II of the OT and the striatal bridges (red arrow). E14-born neurons contribute to more medial VS structures than E15- and E16-born cells, which contribute almost exclusively to the medial islands of Calleja (red arrowheads). Scale bar = 300 μm (A and B), 1 mm (C–H).

To investigate the possibility that neurons migrate in the *Sox1*-null OT regions, but are not visible because they do not express *Sox1* ^βgeo^ and other differentiation markers, we exposed embryos to BrdU. This way, we permanently marked all proliferating precursors independently of *Sox1* or other striatal-marker gene expression and followed them at later embryonic stages and after birth (Figure 4). Labeling the precursors at different embryonic days also provided information on the birthdates of the OT neurons, which were previously unknown for the mouse. Specifically, birth of ventral striatal neurons commences early at E13 and continues until birth (Figure 4A, 4C, 4E, and 4G; data not shown) and is consistent with data from the rat [41,42]. Furthermore, in wild-type embryos, BrdU exposure between E13 and E16 with examination of embryos either 72 h later (Figure 4A; data not shown) or after birth (Figure 4C, 4E, and 4G) showed that early-born neurons migrate more laterally than those born later.

In the *Sox1*-null embryos, the presence and distribution of cells labeled with BrdU between E13 and E16 shows that the olfactory cortex is largely normal (bracket in Figure 4D), but in the VS area the number of labeled cells was found to be greatly reduced (Figure 4B, 4D, 4F, and 4H). Furthermore, in the *Sox1*-null postnatal brain the striatal mantle is more densely populated by BrdU-labeled cells (Figure 4D–4H), consistent with the general increase of X-gal staining observed throughout the striatum (see Figure 2). The above data suggest that in the absence of SOX1, early- and late-born neurons fail to migrate to the appropriate position to form the ventral areas of the striatum.

### The Generation and Patterning of LGE Precursors Is Normal in the Absence of SOX1

**Figure 5:**
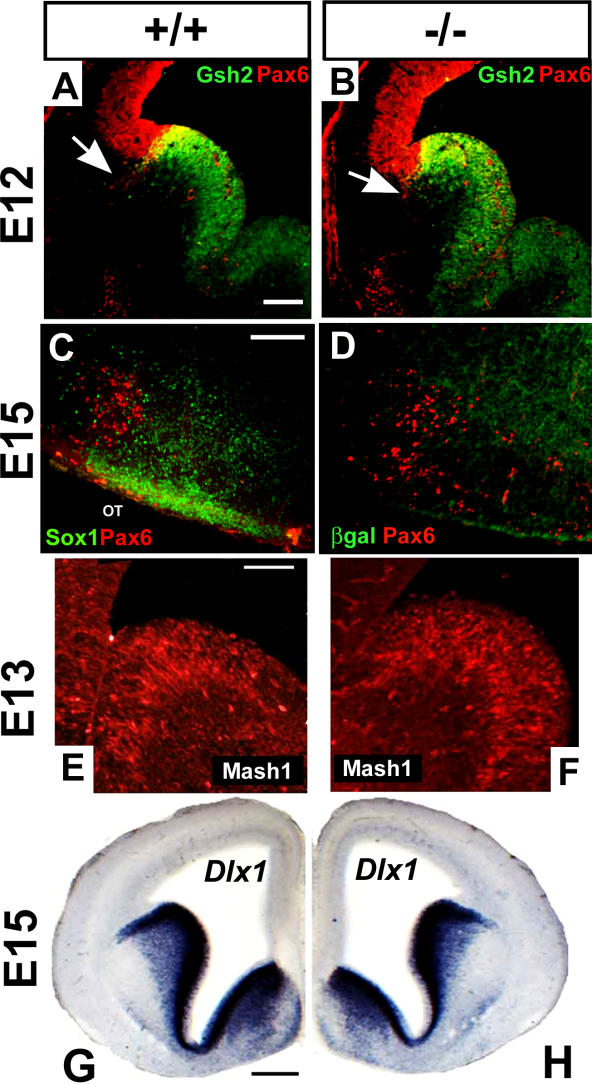
Normal Generation and Patterning of LGE Precursors in the Absence of SOX1 (A and B) Immunocytochemistry and on coronal brain sections of dorsal and ventral telencephalic markers in wild-type (+/+) and *Sox1*-null (−/−) embryos. PAX6 and GSH2 immunocytochemistry in the dorsal LGE at E12 shows no difference at the expression boundary in the absence of SOX1; the arrows point at the stream of PAX6-positive cells emanating from the boundary. Double immunostaining for SOX1/PAX6 in wild-type brain (C), and for β-galactosidase/PAX6 (D) in the *Sox1* ^βgeo/−^ brain, at the VS area at E15. Note the presence of the PAX6-positive neurons in the area of the VS in the *Sox1* ^βgeo/−^ brain. (E and F) MASH1 immunocytochemistry in the LGE of wild-type (E) and *Sox1*-null brain (F), at E13. No changes are detected. (G and H) The distribution of *Dlx1*-expressing cells, as detected by in situ hybridization, is similar in both wild-type and mutant brains. Scale bar = 300 μm (A and B), 200 μm (C–F), 150 μm (G and H).

It is known that the majority of VS neurons derive from precursors that are born in the LGE [10,15,43]. To investigate whether the defect is in the patterning of precursors, we examined the expression pattern of various transcription factors that mark LGE progenitors and are known to have a role in OT neuronal specification (Figures 5, S1, and S2).

The homeodomain transcription factors PAX6 and GSH2 are expressed, respectively, in the pallial and subpallial precursor domains of the dorsal LGE. The boundary between them has been shown to be essential for the patterning of VS precursors [10,11]. Specifically, GSH2-null mice do not form early-born OT neurons, and the precursors of the dorsal LGE are lost as PAX6 expands ventrally into the LGE. However, loss of both PAX6 and GSH2 restores dorsoventral patterning and partially rescues OT formation [9–11]. Using double antibody immunohistochemistry in embryos at E12–E16, we found that loss of SOX1 has no effect on the expression of GSH2/PAX6 and the boundary in the dorsal LGE (Figures 5A, 5B, and S1). In addition, at this boundary, PAX6-expressing postmitotic cells form a stream (arrow in Figure 5A) that extends laterally to the VS (Figures 5A, 5C, S2E, and S2F) [6,7,44]. In the absence of SOX1, the stream of PAX6-positive postmitotic cells is normal (arrow in Figures 5B and S2F), but to characterize the PAX6- and SOX1-expressing neurons in the region of the OT, we used double antibody immunohistochemistry. Specifically, for the wild-type brain sections we used PAX6 and SOX1 antibodies, but to visualize the *Sox1*-expressing cells in the *Sox1* ^βgeo/M1^-null brain sections we used an antibody for β-galactosidase.

Our data showed that PAX6 and SOX1 proteins were co-expressed in progenitors (Figures S2E and S2F), but in postmitotic cells of the LGE this expression became mutually exclusive (Figure 5C and 5D). *Pax6*-expressing neurons were clustered laterally to those expressing *Sox1,* at the border between the OT and olfactory cortex (Figures 5C, S2A, and S2C). In *Sox1*-null mice, the postmitotic *Pax6-*expressing cells were distributed throughout the VS area (Figures 5D, S2B, and S2D), suggesting structural disorganization. It is unlikely that these ectopically localized *Pax6*-expressing neurons are mis-specified *Sox1*-null neurons, because they should be expressing both PAX6 and β*geo* from the mutant *Sox1* ^βgeo^ allele, but they do not (Figure 5D).

The LGE structure consists of neural progenitors with radial glial characteristics, having fibers that extend from the VZ to the pial surface. These cells also provide the substrate for the migration of neurons [45,46]. Staining with X-gal (see Figure 1) and β-galactosidase antibody in mice carrying the *Sox1* ^βgeo^ allele allowed visualization of the cytoplasmic compartment of the SOX1-expressing progenitors, which have radial glial morphology (Figures S2F and S3). We examined the morphology of radial glia in the *Sox1*-null LGE using the RC2 antibody [47], but we did not find any difference from wild-type (Figure S4). Therefore, we conclude that the abnormal distribution of *Pax6*-expressing neurons in the LGE of the *Sox1*-null mice is unlikely to be caused by abnormal morphology of the radial glial fibers or substantial loss of radial glia-like precursors.

In the ventral telencephalon, the bHLH transcription factor MASH1 [18] and the homeodomain factor DLX1 mark LGE precursors [8]. Ablation of MASH1 in mice causes loss of specific subpopulations of precursors and striatal neurons that contribute to the OT and nucleus accumbens [18]. In addition, *Gsh2*-null mice, which also fail to develop the OT, exhibit reduced *Dlx1* expression in LGE precursors [9]. We therefore examined the expression of these two genes in *Sox1*-null embryos, but found no difference (Figure 5E–5H), indicating that there is no deficit of early or late LGE precursors in the absence of SOX1. Collectively, the above data show that SOX1 is not required for patterning, generation, and maintenance of LGE precursors.

### *Sox1* Expression from the Endogenous *Sox2* Promoter Can Be Tolerated In Vivo

**Figure 6:**
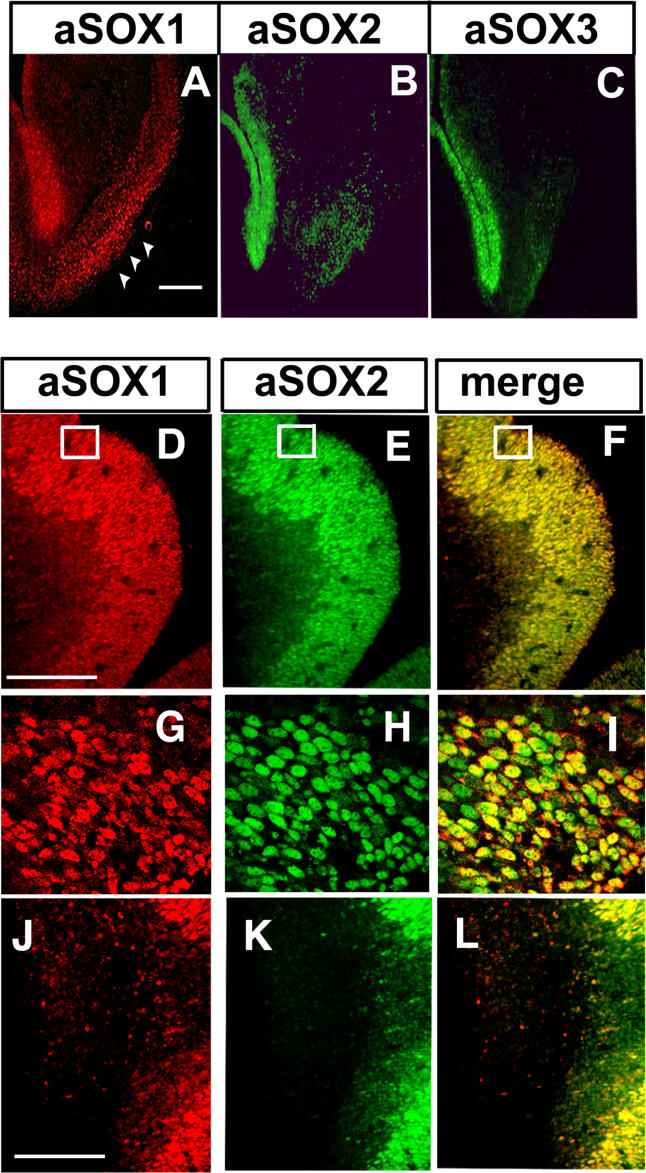
SOX2 and SOX3 Down-Regulation in LGE Neurons and SOX1/SOX2 Co-Expression in LGE Precursors Immunofluorescence of coronal sections at LGE levels in (A–C) E15- and (D–L) E13-stage wild-type embryos visualized on a confocal microscope: antibody staining for (A, D, G, and J) SOX1 (red), (B, E, H, and K) SOX2 (green), (C) SOX3 (green), (D–L) double SOX1 (red) and SOX2 (green), and (F, I, and L) merged. In the OT area and the LGE mantle, there are more neurons expressing SOX1 (A and J) than SOX2 (B and K) and SOX3 (C). Note the extensive co-expression of the SOX1 and SOX2 in precursors (D–I). (G–I) are higher magnifications of the areas within the rectangles. Scale bar = 300 μm.

It has been shown that all three *SoxB1* genes are expressed [25,27] and share similar functions in neural precursors [31,32]. However, in the postmitotic cells of the mantle and the VS area, antibody staining for each of the genes at E15 indicated that SOX2- and SOX3-positive neurons represent a very small population compared to that expressing SOX1 (Figure 6A–6C). Therefore, it is likely that the other *SoxB1* genes compensate for the loss of *Sox1* in precursors, whereas they cannot do so in the LGE postmitotic cells. Nevertheless, it remained unknown whether SOX1 functions solely in precursors for VS fate specification or in postmitotic cells for maintaining this fate and the emergence of specific subtype identity and migration. To address this, we generated mice that express *Sox1* mainly in precursors and not in LGE neurons. We took advantage of the fact that *Sox2* is co-expressed with *Sox1* in precursors but it is down-regulated in LGE neurons, and generated mice that express *Sox1* from the endogenous *Sox2* promoter. We confirmed the overlap of *Sox1* and *Sox2* expression in the VZ/SVZ of the LGE by staining serial coronal telencephalic sections with antibodies for each of the two genes and counter-staining with the nuclear stain TOTO at E14 (Figure S5), and by performing double anti-SOX1 and -SOX2 immunohistochemistry at E13 (Figure 6D–6L).

**Figure 7:**
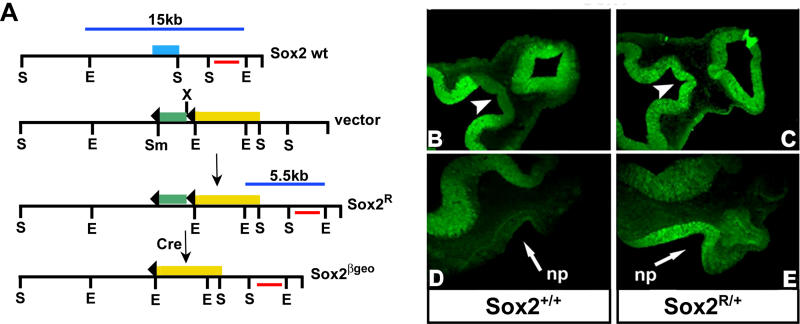
The *Sox2* ^R^ Allele Delivers SOX1 in *Sox2*-Specific Expression Sites (A) Strategy for targeted replacement of the SOX2 coding region with that of SOX1 and IRES-βgeo. Restriction enzymes: S, SalI; E, EcoRI; Sm, SmaI; X, Xho. Green boxes indicate *Sox1*; black arrowheads indicate *LoxP* sites; yellow boxes indicate IRES βgeo; blue lines indicate fragments appearing in Southern blots of EcoR1-digested genomic DNA, hybridized with the external probe, which is shown with red lines. Black arrows show the locus after recombination, homologous and Cre-mediated where is indicated. (B–E) SOX1 immunostaining of frontal sections from E10 embryos. (B and D) Sox2^+/+^ and (C and E) *Sox2* ^R/+^ showing the ectopic expression of SOX1 in the diencephalon (arrowheads) and the nasal pit (np) at E13.

The replacement of the SOX2 open reading frame with that of SOX1 was achieved by targeting the *Sox2* allele (Figure 7A). The new allele, *Sox2* ^R^, was engineered to express not only *Sox1* but also β*geo* via an internal ribosomal entry site (IRES). Furthermore, the coding region of SOX1 in the *Sox2* ^R^ allele was flanked by *LoxP* sites, which can be deleted using Cre-mediated recombination [48]. In this way, we generated a mouse line carrying another allele, termed *Sox2* ^βgeo2^ (but referred to hereafter as *Sox2* ^βgeo^), which expresses only the β*geo* reporter gene from the *Sox2* promoter (Figure 7A) and not SOX1. Like the *Sox2* ^βgeo/+^ heterozygotes, *Sox2* ^R/+^ mice were viable, fertile, and phenotypically normal, indicating that SOX1 over-expression in precursors, as well as ectopic expression in other locations where *Sox2* is uniquely expressed, does not cause any obvious developmental abnormality.

X-gal staining of embryos showed that both targeted *Sox2* alleles (*Sox2* ^βgeo^ and *Sox2* ^R^) express β*geo* in the CNS, but to verify that SOX1 protein was produced from the *Sox2* ^R^ allele, we used SOX1 antibody staining. We found that SOX1 protein was ectopically present at sites where SOX2 normally shows unique expression—for example, in the floor plate of the diencephalon (arrowheads in Figure 7B and 7C) and in the sensory placodes (arrows in Figure 7D and 7E). The intensity of the immunostaining at ectopic sites was comparable to the staining in areas with expression from two wild-type *Sox1* alleles (VZ/SVZ), indicating that the level of expression was similar to the wild-type allele. Therefore, the *Sox2* ^R^ allele produces SOX1 ectopically in all neurons uniquely positive for SOX2 and increases the endogenous level of SOX1 in precursors and neurons that express both genes, without causing an obvious defect in mice. The fact that ectopic expression does not cause any obvious phenotype suggests either that the partner factors required for SOX1 target specificity [19] are absent in those cells uniquely expressing *Sox2* or that the two proteins are interchangeable, sharing target genes. Further experiments are required to clarify this.

### *Sox1* Over-Expression in Precursors Does Not Increase VS/OT Neuronal Fate Specification

**Figure 8:**
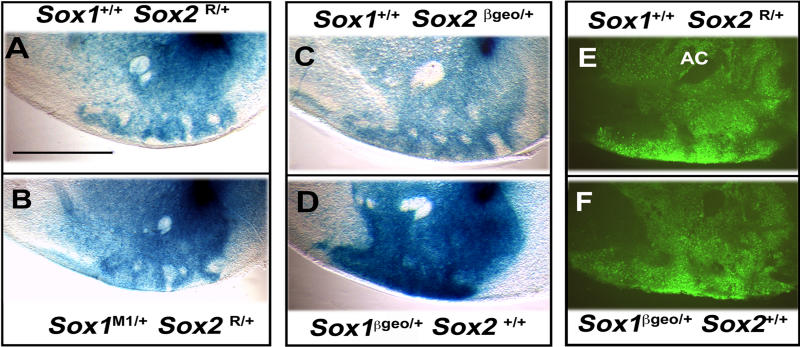
The Distribution of VS Neurons Is Unaffected in Mice Over-Expressing SOX1 from the *Sox2* ^R^ Allele (A–D), X-gal staining of coronal sections from the ventral telencephalon of P0 mice. Note that there is no difference in the distribution of *Sox2*-expressing OT neurons with SOX1 (A) or without SOX1 (C), and in *Sox2* ^R/*+*^ mice with two wild-type *Sox1* alleles (A) or one (B). Comparison of the number and distribution of neurons expressing *Sox2* ^βgeo^ in (C) and *Sox1* ^βgeo^ in (D) shows overlapping expression. (A–C) show 150-μm sections, and (D) shows a 80-μm section. (E and F) DARPP-32 immunostaining of coronal sections from the ventral telencephalon of P10 *Sox2* ^R/*+*^ and *Sox1* ^βgeo/+^ single heterozygous mice, showing no difference in the generation and migration of OT neurons. AC, anterior commissure. Scale bar = 100 μm.

To investigate more subtle defects due to the over-expression of *Sox1* in precursors of the *Sox2* ^R/+^ mice, we examined several litters (*n* > 10) of mice and visualized the migration of the *Sox1*/*Sox2*-positive neurons in the VS via X-gal staining. Newborn mice carrying *Sox2* ^R/+^ with two (*Sox2* ^R/+^, *Sox1* ^+/+^; Figure 8A) or one (*Sox2* ^R/+^, *Sox1* ^M1/+^; Figure 8B) *Sox1* endogenous wild-type alleles were compared with those carrying *Sox2* ^βgeo/+^, *Sox1* ^+/+^ that do not express *Sox1* ectopically (Figure 8C). All the above mice have only one wild-type *Sox2* allele. We also compared the *Sox1*-β*geo* and *Sox2*-β*geo* neurons of the ventral telencephalon in *Sox1* ^βgeo/+^ (Figure 8C and 8D) and *Sox2* ^R/+^ (Figure 8A) mice, respectively. In this area of the brain, the *Sox2*-positive neurons are far fewer than those positive for *Sox1.* Therefore, for comparison purposes, we used thin tissue sections (80 μm) with short X-gal staining (3 h) for *Sox1*-β*geo,* and thicker sections (100–150 μm) with a long staining period (48 h) for *Sox2-*β*geo*. Consistent with the antibody staining data (see Figure 6B), *Sox2-*β*geo* neurons contribute to the OT, indicating that they are a subset of the VS neuronal population. Therefore, the ectopic expression of *Sox1* in LGE neurons is expected to be very limited. More importantly, the migration and the number of LGE neurons expressing β*geo* via the *Sox2* promoter were found to be the same regardless of the number of endogenous *Sox1* alleles or the ectopic presence of *Sox1* (compare Figure 8A, 8B, and 8C). To further investigate the differentiation of the VS neurons, we used the striatal-specific markers dopamine and cAMP-regulated phosphoprotein (DARPP-32) at postnatal stages and found them to be unaffected in *Sox2* ^R/+^ mice (Figure 8E and 8F). The data therefore indicate that the over-expression of SOX1 in precursors does not increase OT neuronal specification.

### *Sox1* Expression in Precursors Cannot Rescue OT Neuron Development

**Figure 9:**
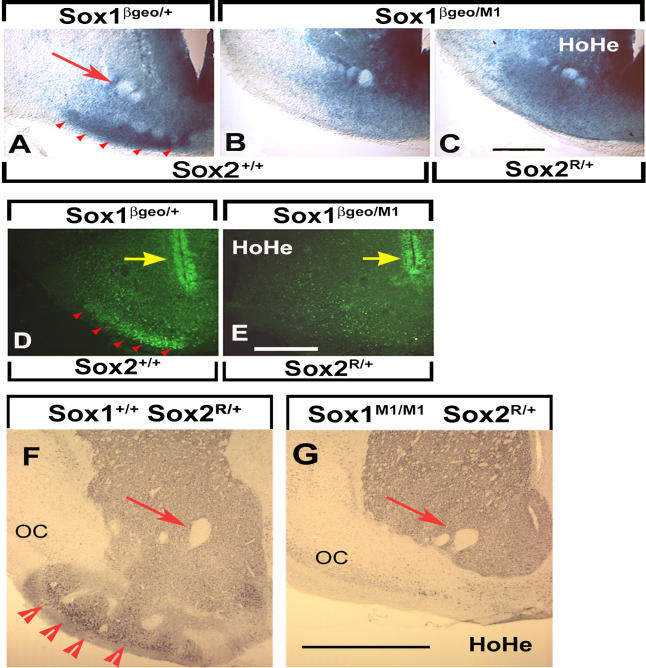
*Sox1* Expression in Precursors Is Not Sufficient for the Emergence of OT/VS Neurons (A–C) X-gal staining of coronal sections from the ventral telencephalon showing *Sox1-*β*geo*-expressing OT-prospective neurons at E16-stage embryos with one wild-type *Sox1* allele, *Sox1* ^βgeo/+^, in (A), and none, *Sox1* ^βgeo/M1^, in (B), and HoHe (*Sox1* ^βgeo/M1^, *Sox2* ^R/+^) in (C). Note the absence of X-gal-stained neurons in the area of the VS (red arrowheads), indicating failure of the *Sox2* ^R^ allele to rescue OT neuron development in the HoHe embryos. Expression of βgeo from the *Sox2* ^R^ allele in the HoHe (C) may account for the slight increase of X-gal staining compared to (B). (D and E) SOX1 immunostaining at E16 embryos performed on the other halves of the brains of (A and C), respectively. Note that the level of SOX1 expression in the precursors (yellow arrows) is the same, whether it is expressed from the *Sox2* ^R^ allele in the HoHe (E) or from one of the *Sox1* wild-type alleles in the *Sox1* single heterozygotes (D). Note in the HoHe (E), this expression is not sufficient for the development of OT neurons. (F and G) DARPP32 immunostaining of coronal brain sections from *Sox1* ^+/+^ *Sox2* ^R/+^ (F) and *Sox1* ^M1/M1^ *Sox2* ^R/+^ (G) P10 mice indicating loss of VS neurons. Red arrowheads indicate OT; red arrows indicate anterior commissure. OC, olfactory cortex. Scale bar = 500 μm (A–E), 1 mm (F and G).

To address directly whether SOX1 function is essential in precursors, we crossed *Sox1* ^M1/+^, *Sox2* ^R/+^ mice with *Sox1* ^βgeo/+^, *Sox2* ^+/+^ mice and examined whether offspring carrying *Sox2* ^R/+^ without any wild-type *Sox1* alleles (*Sox1* ^M1/βgeo^) could develop OT. In these *Sox1* ^R/+^ embryos that carry no endogenous *Sox1* functional allele (termed here HoHe), SOX1 is expected to be expressed only via the *Sox2* ^R^ allele in precursors and become down-regulated in postmitotic LGE cells. However, β*geo* expression from the endogenous *Sox1* mutant allele marks precursors and OT prospective neurons. We followed the *Sox1* ^M1/βgeo^ prospective OT neurons with X-gal to determine whether they were capable of contributing to the OT in HoHe embryos (Figure 9). The *Sox2* ^R^ allele also expresses β*geo*; however, in LGE postmitotic cells, *Sox2*-β*geo* expression is much less than that of *Sox1-*β*geo* and is not very visible by short (3 h) X-gal staining (only a slight increase of X-gal staining is seen in the VS; Figure 9C compared to Figure 9B).

Each brain was split into left and right hemispheres, and coronal sections of the left were used for short staining with X-gal (Figure 9A–9C) whereas sections of the right were stained with SOX1 antibody (Figure 9D and 9E). The hemispheres stained for X-gal showed characteristic staining of OT neurons in heterozygous *Sox1* ^βgeo/+^ mice (red arrowheads in Figure 9A), but the hemispheres of the *Sox1*-null embryos (*Sox1* ^βgeo/M1^) with *Sox2* ^R/+^ (Figure 9B) or without (Figure 9C) did not. This indicates that *Sox1*-null embryos do not develop OT despite the presence of the *Sox2* ^R^ allele and SOX1 protein in progenitors. To verify the presence of SOX1 protein in the precursors of the HoHe mice, we examined the other hemisphere that was stained with SOX1 antibody. In the *Sox1* ^βgeo/+^ embryos, we found SOX1 present in the VZ (yellow arrows in Figure 9D and 9E) and the OT neurons (red arrowheads in Figure 9D). In *Sox1* ^βgeo/M1^ (null) embryos, SOX1 expression was completely absent (data not shown); in the HoHe embryos, SOX1 protein was present in the VZ (yellow arrow in Figure 9E) and in very few neurons of the LGE (Figure 9E). HoHe mice, like the *Sox1*-nulls, are born with small eyes, and around weaning age develop seizures associated with lethality, which, if anything, is increased compared to that of *Sox1*-null mice (data not shown). In the brain of P10 HoHe mice, we used staining with DARPP-32 antibody (which is a SOX1-independent striatal marker) to investigate the recovery of OT neurons, and found staining in the striatal mantle but not in the VS (Figure 9G compared to Figure 9F). We therefore concluded that SOX1 expression in precursors is not sufficient to rescue VS/OT neuron fate specification, and that the continued presence of SOX1 in postmitotic cells is required for their identity.

### *Sox1/Sox2* Expression in Neurons Is Sufficient for Their Migration to the VS

**Figure 10:**
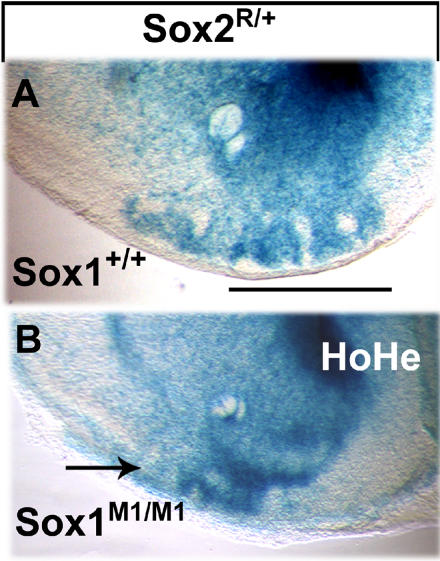
*Sox1* Expression in Postmitotic LGE Cells Is Sufficient for Neuronal Migration in the VS X-gal staining of coronal sections from the ventral telencephalon of P0 mice indicating the migration of neurons expressing SOX1 from the *Sox2* ^R^ allele in the presence (A) and absence (B; HoHe) of endogenous *Sox1* wild-type genes. Black arrow points at the striatal bridges forming in HoHe mice. Scale bar = 100 μm.

We have shown that in mice carrying two (*Sox1* ^+/+^), or one (*Sox1^M1^* ^/+^), *Sox1* wild-type alleles (see Figure 8A, 8B, and 8C), the migration of the *Sox2-*positive LGE neurons is not overtly different from that observed in mice carrying the *Sox2* ^R/+^ allele. However, it remained unknown whether the *Sox1*/*Sox2* double-positive LGE neurons migrated to the VS when both *Sox1* endogenous alleles were missing (HoHe mice). We used X-gal staining to follow these neurons in several litters (*n* > 10), including HoHe mice, which have two *Sox1* ^M1^ alleles and thus β*geo* expression exclusively driven by the *Sox2* ^R^ allele. We found that in the LGE of these mice, the double-positive neurons are generated and migrate to the OT area (compare Figure 10A and 10B), but this area is compacted in the absence of the majority of the OT/SOX1 neurons. The above data show that the continued expression of *Sox1* in neurons of the LGE is sufficient to direct their migration to the OT in the absence of endogenous *Sox1*.

## Discussion

The specification of neurons in the ventral telencephalon has been shown to depend on several transcription factors that are expressed mainly in proliferating precursors. However, it was unknown to what degree specification in precursors included the emergence of neuronal subtype identity in the ventral telencephalon, and whether expression of additional transcription factors was required. We showed that the differentiation and migration of early- and late-born neurons that constitute the VS require SOX1 expression not only in precursors but also in postmitotic cells. Furthermore, in this region, the migration and organization of other neurons such as those expressing *Pax6* also depend on the presence of SOX1-positive VS neurons. The finding that SOX1 functions in neurons to control migration and identity is novel and suggests that the other SOXB1 factors, in addition to their roles in precursors, have similar functions in neurons.

### Identity and Migration of Neurons in the VS

The development of subtype identity and migration of neurons in the ventral telencephalon has not been well characterized. The expression of differentiation markers reveals neurons in both VS and dorsal striatum, but we showed that SOX1 specifically marks a large population of VS neurons that form the principal layer II of the OT, the islands of Calleja, and the nucleus accumbens (see Figures 1 and 2). In addition, we showed that the neurons expressing *Sox1* are born continuously from E13 until the first postnatal week and that these migrate to a ventrolateral region of the telencephalon, with later-born neurons positioned progressively to more medial positions (see Figure 4). In the absence of SOX1, the majority of neurons of the VS fail to develop. All *Sox1*-expressing neurons of the OT and the islands of Calleja require SOX1 for their development, but it is essential only for the shell of the nucleus accumbens, although the core also expresses it. While neurons of the adjacent striatal mantle and the olfactory cortex that do not express *Sox1* develop normally in its absence, other groups of neurons within the VS appear to be disorganized. Specifically, we identified a distinct population of neurons located lateral to the OT at the border with the olfactory cortex that expresses *Pax6,* but not *Sox1*. In the absence of SOX1, these neurons migrate into more medial positions, occupying the space of the missing OT neurons (see Figure 5). These are not mis-specified *Sox1*-null neurons because they do not express β*geo*. This indicates that *Sox1*-expressing OT neurons play a non-cell-autonomous role in the organization of other neurons in this region, including the production of essential signals for migration. Most likely, the disorganization of the VS in the absence of SOX1 results in abnormal local neuronal connectivity, which in turn leads to the abnormal (epileptiform) electrophysiological behavior observed in the SOX1-deficient animals [34].

### SOX1 Function in Precursors

SOXB1 factors share considerable homology in both their DNA binding and C-terminal transcriptional activation domains, and they are co-expressed in precursors. It is therefore possible that in the LGE precursors, the role of SOX1 in the specification of OT/VS neurons is redundant. However, as *SoxB1* genes have a broad expression in the neuroepithelium, we have to assume that their specific function at different areas of the VZ, and particularly the VZ of the LGE, is controlled by the presence of LGE-specific partner factors. *SoxNeuro* and *Dichaete*, the two *Drosophila* orthologs of the vertebrate *SoxB1* group genes, also show overlapping functions during neural development [49]. Furthermore, in *Drosophila,* these two genes have been shown to genetically interact with the dorsoventral patterning genes *ind (intermediate neuroblast defective)* and *vnd (ventral nerve chord defective)* [50,51]. The vertebrate orthologs of *ind* and *vnd* are *Gsh1*/*2* [52,53] and *Nkx2.2* [54], respectively. In the mouse, *Gsh2* is expressed in the VZ/SVZ, and like *Sox1,* its loss results in a reduction of VS neurons. As target gene specificity of SOX proteins depends on partnering with other transcription factors [20], our work, along with the data from *Drosophila,* supports the hypothesis that in the LGE precursors GSH1/2 may act as partners for SOXB1 factors to initiate ventral telencephalic neuronal identity.

The neurons of the VS area occupy approximately a quarter of the striatal mass [55], and migrate there over a period of at least 10 d (E13 to first postnatal week). The LGE precursors that generate the OT/VS in the LGE are expected to have an equivalent representation during this period of development. In *Gsh2*-null mice, which are missing early-born OT neurons, there is a deficit of precursors in the LGE, readily seen by the reduced expression of *Dlx1*/*2* in LGE precursors [9–11]. In *Sox1*-null mice, the neuronal deficit is more severe than that in *Gsh2* mutants, as it includes both early- and late-born OT/VS neurons. However, BrdU labeling and LGE precursor-specific marker analysis, including *Gsh2, Dlx1*/*2, Mash1,* and *Pax6,* in *Sox1*-null brains at different stages did not show any deficit in precursors. The increase of X-gal-stained (*Sox1-*β*geo*; see Figure 2) and BrdU-labeled neurons (see Figure 4D and 4F) in the area of the septum and the striatum supports the notion that the VS/OT neurons are born but lack VS subtype identity to migrate toward ventral positions. The normal expression of TuJ1, a marker of immature neurons, excluded the possibility that loss of SOX1 delays or enhances differentiation. Therefore, in the absence of SOX1, the precursors are there and generate neurons, but these fail to migrate to the VS because they assume different identity and position. The finding that *Sox1*-null neurons contribute widely to different areas argues that the presence of SOX1 provides neurons with ventral identity and the ability to migrate to ventral regions.

### Emergence of VS Neuron Identity

To test the role of *Sox1* expression in neurons and to determine whether ventral identity emerges in postmitotic cells, we limited expression of *Sox1* largely to precursors of LGE neurons. *Sox2* ^R/+^ mice express *Sox1* from one of the *Sox2* alleles. When the *Sox2* ^R^ allele is present in animals with no endogenous *Sox1* wild-type alleles (HoHe), SOX1 expression mimics that of SOX2—being present in VS/OT precursors but largely absent from the neurons they give rise to. HoHe mice also fail to develop the majority of VS/OT neurons (see Figure 9) and exhibit an equally severe phenotype to that of *Sox1*-null mice in the OT. As these mice reproduce faithfully the *Sox1*-null phenotype without any evidence of a partial rescue, it is unlikely that this is the result of incomplete expression of *Sox1* from the *Sox2* promoter in the precursors. However, to exclude the possibility that the failure of OT/VS neuron development in HoHe mice was due to a low level of expression of SOX1 protein in precursors, we used one hemisphere of the brain to assay OT development and the other for SOX1 antibody staining, linking in each animal the phenotype with the presence of SOX1 protein in precursors. We found no difference in the extent and level of expression of SOX1 protein in the VZ/SVZ of embryos with one copy of *Sox1,* whether it is expressed from the *Sox2* locus in HoHe (see Figure 9E) or the endogenous *Sox1* allele in *Sox1* ^βgeo/+^ heterozygotes (see Figure 9D). Therefore, the emergence of VS/OT identity requires *Sox1* expression in postmitotic cells. Consistent with the above findings, the small population of LGE postmitotic cells in HoHe mice that maintain SOX1 expression from the *Sox2* ^R^ allele migrate to the VS. However, the number of these neurons is small and cannot rescue the deficit in the area of the VS. In conclusion, although specification of neuronal identity is initiated in precursors, emergence of neuronal subtype and ventral migration require the continued presence of SOX1. Our findings suggest that in other brain areas, subtype identity and migration may also be controlled by the expression of transcription factors in postmitotic cells.

The current study, along with our previous one showing that SOX1 expression in the lens of the mouse is responsible for terminal differentiation and the expression of γ-crystallin genes [37], has revealed that SOX1 has important functions in postmitotic cell differentiation at two distinct sites. It is possible that the other SOXB1 factors have similar roles in postmitotic cells in which their expression is maintained.

## Materials and Methods

### 

#### Gene targeting.

The β*geo* gene was inserted into the *Sox1* single exon as previously described [37]. The resulting targeted locus produces a fusion protein consisting of the first 50 amino acids from SOX1 (which excludes the HMG box) followed by ten amino acids that are encoded by a synthetic linker sequence, and the βgeo sequence (including a polyadenylation signal). Tissue culture was carried out as described before [37], omitting the addition of gancyclovir for negative selection. The targeting vector did not contain any other selectable markers or promoters, and the targeting frequency was 1/52. As *Sox1* is not normally expressed in embryonic stem (ES) cells, we used the minimum level of G418 for selection. Positive recombinants were identified by Southern blotting, using a 3′ 1-kb external probe on an EcoRI digest. Three ES cell clones were obtained, and one was successfully passed through to the germ line. All anatomical investigations were performed on mice of mixed genetic background. *Sox1* ^βgeo/+^ mice were mated with mice that were heterozygous for the previously described [37] *Sox1* deletion (*Sox1* ^M1^) and did not express β-galactosidase.

For the *Sox2* replacement vector, the 5′ and 3′ homologies used were the same as described before [30]. A SmaI*-*XhoI 2.2-kb *Sox1* fragment containing the SOX1 open reading frame was flanked by *LoxP* cassettes followed by the NotI-SalI IRES–βgeo-polyA fragment (plasmid gift from Dr. A. Smith, University of Edinburgh). The replacement vector was linearized with SalI and electroporated into ES cells. Positive recombinants were identified by Southern blotting as described before [30]. Several targeted ES cells were isolated at a frequency of 1/20 and gave germ-line transmission of the mutation. Heterozygous animals carrying the *Sox2^Sox1^* ^βgeo^ allele (referred to in the text as *Sox2* ^R^) expressed *Sox1* and β*geo* where *Sox2* is normally expressed.

Deletion of the SOX1 coding region from the *Sox2^Sox1^* ^βgeo^ allele was achieved via pronuclear injection of a supercoiled plasmid expressing Cre-recombinase (gift from Dr. K. Rajewsky, Harvard Medical School). Although in the text we refer to this new allele as *Sox2* ^βgeo^, it is officially named *Sox2* ^βgeo2^ to distinguish it from the one previously described [30].

#### X-gal staining and in situ hybridization.

For β-galactosidase staining, fetal, newborn, or adult mouse brains were processed as previously described [30]. Detection of *Sox1* mRNA was performed in whole embryos, as described previously [27,34]. The probes that were used on embryonic brain slices were generated by RT-PCR from embryonic brain cDNA. The position of the probes was *Dlx1* (nt 41–573), *Brn4* (nt 199–541), and *Robo* (nt 8–779). All fragments were cloned into a suitable cloning vector (pGET-easy, Promega, Madison, Wisconsin, United States), and were re-amplified using a sense oligonucleotide and an oligonucleotide upstream of either the T7 or SP6 sites. The resulting products were gel-purified, and 40 ng was used for probe synthesis. The brains were processed as described before [27,34].

#### BrdU labeling

A 25 mg/ml solution of BrdU (Sigma, St. Louis, Missouri, United States) was made in PBS warmed to 37 °C. The solution was sterilized through a 0.2-μm syringe filter and injected into the peritoneal cavity of pregnant mice (0.1 ml per 25 g of body weight, to give a final dosage of 0.1 mg/g). Brains were harvested 1 or 72 h after the injection, or on P16. The brains were fixed in 4% PFA in PBS at 4 °C overnight, embedded in paraffin wax, and cut into 5-μm thick sections. The sections were processed for immunohistochemistry as previously described [18].

#### Immunohistochemistry.

The source of antibodies and the dilutions used are as follows: PAX6, 1:10 (gift from Dr. J. Briscoe); GSH2, 1:5,000 (gift from Dr. K. Campbell); β-galactosidase 1:2,000 (Cappel); TuJ1, 1:1,000 (Novus Biologicals, Littleton, Colorado, United States); and SOX1, 1:500; SOX2, 1:500; and SOX3, 1:500 (gift from Dr. T. Edlund). MASH1, 1:2 (gift from Dr. F. Guillemot) [18], RC2 [56], and DARPP-32 [57] were used as previously described. For single or double immunofluorescence, embryonic tissue was fixed either for 1 h in 4% PFA in PBS at room temperature or for 15 min in MEMFA as described before [58]. The brains were then washed in PBS, cryoprotected overnight in 30% sucrose in PBS at 4 °C, embedded in OCT (Raymond Lamb), and cut in 10-μm and 15-μm sections using a cryostat. Sections were rehydrated in PBS, blocked in 4% goat serum and 0.1% Triton X-100 in PBS, and incubated overnight at 4 °C with primary antibodies diluted in the blocking solution. After incubation, the slides were washed in PBS and incubated with fluorescent secondary antibodies (FITC- or TRITC-labeled, 1:200 in blocking solution) for 1 h at room temperature.

Slides for double immunolabeling were first immunostained for SOX1 as described above and visualized with Alexa568 goat anti-rabbit antibody (1:500, Molecular Probes, Eugene, Oregon, United States), and then incubated with unlabeled anti-rabbit secondary antibody (1:100, Dako, Glostrup, Denmark) for 1 h at room temperature to block existing unlabeled anti-SOX1 antibody. Subsequently, the slides were incubated with anti-SOX2 antibody in blocking solution without Triton X-100 for 48 h at room temperature and visualized with Alexa488 goat anti-rabbit antibody (1:500, Molecular Probes). The cross-reactivity of the SOX1 and SOX2 antibodies when using immunohistochemistry was excluded by looking at tissues where SOX1 (lens; [37]) or SOX2 (see Figure 7B–7E) are uniquely present, and in Western blots where each antibody recognizes a different size band (data not shown). After incubation with the secondary antibodies, the slides were washed in PBS and the sections were mounted with Vectashield mounting medium (Vector Laboratories, Burlingame, California, United States) and observed under either a fluorescent or a confocal microscope.

## Supporting Information

Figure S1 The GSH2/PAX6 Boundary Is Unaffected throughout Development in the Absence of SOX1 Similar to earlier stages (E12; see Figure 5), the expression of GSH2 (green) and PAX6 (red) protein in wild-type (A, C, and E) and mutant brains (B, D, and F) is the same at E14 and E15, as shown by DAPI (blue) nuclear stain. Cx, cortex; lge, lateral ganglionic eminence; mge, medial ganglionic eminence. Scale bar = 200 μm for (C) and (D).
(10 MB TIF).

Figure S2 Abnormal Distribution of VS *Pax6*-Expressing Neurons in the Absence of SOX1 Ventral telencephalic region of coronal brain sections stained with antibodies: PAX6 (brown) at E16 (A and B) and adult (C and D); SOX1 (green) and PAX6 (red) at E14 (E); and SOX1 and β-galactosidase (green) at E15 (F). Note *Pax6*-expressing cells are excluded from the OT region in the ventral telencephalon in wild-type (*Sox1* ^+/+^) mice but not in the mutant*(Sox1* ^−/−^). Arrows indicate ectopically localized *Pax6*-expressing cells. PAX6 and SOX1 are co-expressed in precursors but not in postmitotic cells in both mutant and wild-type. Scale bar = 500 μm for (C) and (D).
(10 MB TIF).

Figure S3 *Sox1*-Expressing VZ Precursors Have Radial Glial Morphology Detail from immunofluorescence with β-galactosidase antibody of an E15 coronal brain section from mice carrying the *Sox1* ^βgeo^ allele. In the cortical ventricular zone, this staining reveals the cytoplasmic compartment of the SOX1-expressing progenitors, which have radial glial morphology.
(3.34 MB TIF).

Figure S4 The Distribution of Radial Glia in the LGE Is Unaffected in the Absence of SOX1 Coronal brain sections of wild-type (+/+) and *Sox1*-null (−/−) mice at E16. Immunostained with RC2 antibody (a radial glia marker) showing no differences.
(2.5 MB TIF).

Figure S5 Widespread Presence of SOX1 and SOX2 Proteins in Nuclei of the LGE VZ Immunofluorescence (green) of E14-stage wild-type coronal brain sections at the level of the LGE stained with SOX1 (C and G) and SOX2 (D and H) antibodies and visualized under a confocal microscope. (A, B, and E–H) are stained with TOTO red-fluorescent nuclear stain. (C, E, and G) and (D, F, and H) show high magnification of the area indicated in the rectangle in (A) and (B), respectively.
(10 MB TIF).

Table S1 No Difference in the Number of LGE Dividing Precursors in the Absence of SOX1 BrdU-positive cells were counted using the Openlab image analysis program (Improvision, Coventry, United Kingdom). Measurements were performed in the area of the LGE (VZ/SVG) and of the pallial VZ. All data are represented as mean ± standard error of the mean. Cell counts were done in at least three different slides (sections) from each brain and in at least three separate optical fields in each slide (*n* = 4). To correct for tissue thickness and to obtain a better estimate of the proliferation within the LGE VZ/SVZ, the numbers of BrdU-positive cells were expressed as a ratio of the total number of LGE VZ/SVZ cells that were counted after hematoxylin staining, and as a ratio to the BrdU-positive cells of the pallial VZ/SVZ. Comparisons were made between wild-type and mutant mice using the unpaired Student's *t*-test (*p* < 0.05).
(21 KB DOC).

### Accession Numbers

The GenBank (http://www.ncbi.nlm.nih.gov/Genebank/) accession numbers for the entities discussed in this paper are *Brn4* (NM 008901), *Dlx1* (NM 010053), *Robo* (MMU 17793), *Sox1* (MN 009233), and *Sox2* (MN 011443).

## Abbreviations

bHLH: basic helix-loop-helix
BrdU: 5-bromo-2′-deoxyuridine
CNS: central nervous system
DARPP-32: dopamine and cAMP-regulated phosphoprotein
E[number]: embryonic day [number]
ES: embryonic stem
IRES: internal ribosomal entry site
LGE: lateral ganglionic eminence
OT: olfactory tubercle
P[number]: postnatal day [number]
SVZ: subventricular zone
TuJ1: anti-βIII-tubulin antibody
VZ: ventricular zone
VS: ventral striatum
β*geo*: β-galactosidase-neo
