# Supplementary material for: Neuronal Migration and Ventral Subtype Identity in the Telencephalon Depend on SOX1
Source: PLoS Biol. 2005 May 17;3(6):e186. doi: 10.1371/journal.pbio.0030186 (PMC1110909; doi:10.1371/journal.pbio.0030186)
Supplement: Supplementary file 1 [file pbio.0030186.st001.doc]

### Table S1. There is no Difference in the Number of BrdU-Positive Cells in the Absence of SOX1

|  | BrdU-positive cells | |
| --- | --- | --- |
|  | LGE VZ/SVZ | Pallial VZ |
| Wild-type | 134 ± 20.5 | 116 ± 7.1 |
| Mutant | 131 ± 24.9 (P=0.72) | 117 ± 7 (P=0.79) |

BrdU-positive cells were counted using the Openlab image analysis program (Improvision). Measurements were performed in the area of the Lateral Ganglionic Eminence (ventricular and subventricular zone) and of the Pallial Ventricular Zone. All data are represented as mean ± S.E.M. Cell counts were done in at least three different slides (sections) from each brain and in at least three separate optical fields in each slide (*n*=4). To correct for tissue thickness and to obtain a better estimate of the proliferation within the LGE VZ/SVZ, the numbers of BrdU-positive cells were expressed as a ratio of the total number of LGE VZ/SVZ cells that were counted after hematoxylin staining, and as a ratio to the BrdU-positive cells of the pallial VZ/SVZ. Comparisons were made between wild type and mutant mice using the unpaired *Student’s* t-test (P<0.05).
